# Supplementary material for: Acceptance, Advocacy, and Perception of Health Care Providers on COVID-19 Vaccine: Comparing Early Stage of COVID-19 Vaccination with Latter Stage in the Eastern Region of Saudi Arabia
Source: Vaccines (Basel). 2023 Feb 20;11(2):488. doi: 10.3390/vaccines11020488 (PMC9963779; doi:10.3390/vaccines11020488)
Supplement: Supplementary file 1 [file vaccines-11-00488-s001.zip › vaccines-2159757-supplementary.pdf]

Perceptions and trust among health care providers (HCP) toward acceptance and advocate for newly developed vaccine for COVID-19

You are invited to participate in this study about perceptions and Trust among Health Care Providers (HCP) toward Acceptance and Advocate for Newly Developed Vaccine for COVID-19. All the information is anonymous and confidential. It takes you 3 minutes to complete the survey. Your participation is appreciated

If you accept to participate please press Next

* Required

Section A

1. Gender *

Mark only one oval.

- ( ) Female
- ( ) Male

2. Age *

Mark only one oval.

- ( ) 25 years and below
- ( ) 26 – 35 years
- ( ) 36 – 50
- ( ) above 50 years

3. Education *

Mark only one oval.

- ( ) Diploma
- ( ) Bachelor
- ( ) Post graduate Diploma
- ( ) Master Degree
- ( ) PhD

4. Total years of experience (since graduation) *

Mark only one oval.

- ( ) 5 years and less
- ( ) 6 – 10 years
- ( ) More than 10 years

5. What is your nationality? *

Mark only one oval.

- ( ) Saudi
- ( ) Non-Saudi

6. Occupation *

Mark only one oval.

- ( ) Doctor
- ( ) Dentist
- ( ) Pharmacist
- ( ) Nurse
- ( ) Physiotherapist
- ( ) Laboratory
- ( ) Other: ____________

7. Marital Status *

Mark only one oval.

- ( ) Married
- ( ) Single
- ( ) Divorce
- ( ) Widow

8. Do you have kids? *

Mark only one oval.

- Yes
- No

9. Do you have comorbidity (any chronic disease)? *

Mark only one oval.

- Yes
- No

10. Do you take any medication on regular base? *

Mark only one oval.

- Yes
- No

11. Do you feel you are at a high risk to get COVID infection? *

Mark only one oval.

- Yes
- No

12. Have you taken the newly developed vaccine to immunize yourself against Covid-19? *

Mark only one oval.

- Yes *Skip to question 14*
- No *Skip to question 13*

Skip to question 18

Section B

13. 13. If No, please indicate the reason *

Mark only one oval.

- I was hesitant about taking the vaccine
- I was worried about the possible side effects
- I was infected with covid 19 < 6 months
- I didn't find an appointment yet

Skip to question 18

Section C

14. When did you take the vaccine (month, year) ? *

____

15. Was it one or two shots ? *

Mark only one oval.

- I received only first shot
- I received both shots

16. Did you experience side effects ? *

Mark only one oval.

- Yes
- No *Skip to question 18*

Section D

17. If Yes, What side effects *

Check all that apply.

- [ ] Pain
- [ ] Itching
- [ ] Fever
- [ ] Other: ____________

Section F

18. Do you think there is a possibility of infection or re-infection following vaccination? *

Mark only one oval.

- Yes
- No

19. Have you advised others to take the newly developed vaccine against COVID-19? *

Mark only one oval.

- Yes I have
- No I have not
- I Was hesitant to do so

20. Who did you trust the most for information on vaccination? *

Mark only one oval.

- Leaders
- Policy makers
- Health care providers
- Media
- Influncers
- None
- Other: ____________

21. Do you have allergies to medication, food, or any vaccine? *

Mark only one oval.

- Yes
- No

22. Have you ever had a serious reaction following a vaccination/ or any bad experience? *

Mark only one oval.

- Yes
- No

23. Do you take influenza vaccine annually? *

Mark only one oval.

- Yes
- No

24. Do you think that mode of administration influence acceptance, oral and nasal are more convenient? *

Mark only one oval.

- Yes
- No

25. Do you think that public hesitancy toward accepting newly developed vaccine is high.? *

Mark only one oval.

- Yes
- No

Section G

Please score 1, 2, 3, 4 or 5 for each statement. (1 reflect strongly disagree, 2 disagree, 3 neutral, 4 agree and 5 strongly agree).

26. I have good medical knowledge on vaccine safety and efficacy. *

Mark only one oval.

- Strongly agree
- Agree
- Neutral
- Disagree
- Strongly disagree

27. I trust the manufacturing country of the vaccine. *

Mark only one oval.

- Strongly agree
- Agree
- Neutral
- Disagree
- Strongly disagree

28. I trust the manufacturing company of the vaccine. *

Mark only one oval.

- Strongly agree
- Agree
- Neutral
- Disagree
- Strongly disagree

29. I believe vaccines is tested long enough for safety and efficacy. *

Mark only one oval.

- Strongly agree
- Agree
- Neutral
- Disagree
- Strongly disagree

30. I think leaders and policy makers should take vaccination first. *

Mark only one oval.

- Strongly agree
- Agree
- Neutral
- Disagree
- Strongly disagree

31. I think previous bad scientific history increases hesitancy e.g (Guillain-Barre Syndrome). *

Mark only one oval.

- Strongly agree
- Agree
- Neutral
- Disagree
- Strongly disagree

32. I think media has created positive impression about the vaccine. *

Mark only one oval.

- Strongly agree
- Agree
- Neutral
- Disagree
- Strongly disagree

33. I think media has created negative impression about the vaccine. *

Mark only one oval.

- Strongly agree
- Agree
- Neutral
- Disagree
- Strongly disagree

34. I believe vaccine's industry is driven by financial motives and not health interest. *

Mark only one oval.

- Strongly agree
- Agree
- Neutral
- Disagree
- Strongly disagree

35. I think honest scientific facts on vaccine provoke acceptance. *

Mark only one oval.

- Strongly agree
- Agree
- Neutral
- Disagree
- Strongly disagree

36. I believe forced vaccination by authorities provoke hesitancy. *

Mark only one oval.

- Strongly agree
- Agree
- Neutral
- Disagree
- Strongly disagree

This content is neither created nor endorsed by Google.

Google Forms
